# Poorer prognosis of ovarian squamous cell carcinoma than serous carcinoma: a propensity score matching analysis based on the SEER database

**DOI:** 10.1186/s13048-020-00675-y

**Published:** 2020-07-01

**Authors:** Cheng Zhang, Tai Ma

**Affiliations:** 1 Anhui Provincial Cancer Institute, The First Affiliated Hospital of Anhui Medical University, Hefei, China; 2 Department of Oncology, The First Affiliated Hospital of Anhui Medical University, Hefei, China

**Keywords:** Squamous cell carcinoma, Ovarian neoplasms, Prognosis, SEER program

## Abstract

**Background:**

Ovarian squamous cell carcinoma (SCC) is a rare cancer with possible poor survival, however no direct evidence supports this viewpoint and the independent prognostic factors are controversial. Patients with ovarian SCC and serous carcinoma (SC) who were diagnosed between 2004 and 2016 were selected using the recent released SEER database. Propensity score matching was used to balance the characteristics of the two groups. The difference of survival between patients with ovarian SCC and SC was explored using Kaplan-Meier method. Cox regression analyses were performed to further identify the independent prognostic factors among patients with ovarian SCC.

**Results:**

Of 15,286 patients (15,106 SC cases and 180 SCC cases), 304 were identified in the matched cohort (200 SC cases and 104 SCC cases). The overall survival and cause-specific survival for patients with SCC were significantly poorer (*P*_*log-rank*_ < 0.001). The median survival time was 21 months for SCC and 95 months for SC. Patients who underwent bilateral salpingo-oophorectomy with hysterectomy and omentectomy seemed to have a better outcome. In multivariate analysis, older age at diagnosis, larger tumor size, bilateral and FIGO stage IV malignancy were the independent risk factors for poor disease outcome.

**Conclusions:**

The prognosis of ovarian SCC is worse than ovarian SC. Older age at diagnosis, advanced disease stage, larger tumor size and bilateral malignancy are the independent risk factors for poor survival.

## Background

In 2020, there will be about 21,750 new cases of ovarian cancer diagnosed and 13,940 ovarian cancer deaths in the United States [1]. The average lifetime risk of developing ovarian cancer for US women is estimated as 1.3% [2]. Ovarian cancer encompasses a heterogeneous group of malignancies and epithelial cancers account for 90% of all cases [2]. The survival of ovarian cancers varies substantially by histological subtype. The 5-year cause-specific survival for all epithelial cancers is 47%, among which serous carcinoma (SC) has the most aggressive nature (5-year cause-specific survival is 43%) while other subtypes have better outcomes (5-year cause-specific survival ranges from 66 to 82%) [2].

Squamous cell carcinoma (SCC) of ovary is a rare entity that constitutes less than 1% of the total ovarian carcinoma cases [3]. This disease predominantly arises from malignant transformation of mature cystic teratoma (MCT) [4, 5]. Patients with ovarian SCC have been reported to have a worse disease outcome [6]. However only one study [7] enrolled more than 200 cases and apparently reported that the median 2-year and 5-year overall survival (OS) rate was 53.0 and 48.4% for MCT-SCC, respectively. By indirect comparison with the aforementioned survivorship of epithelium ovarian cancers [2], the outcome for ovarian SCC seems to be identical with common subtypes. Thus the concept of poorer prognosis for ovarian SCC is challenged given no direct comparison has been made. In addition, it is believed that the patients with advanced International Federation of Gynecology and Obstetrics (FIGO) stage SCC have a significantly worse outcome [7–9]. The values of other clinical findings and treatment strategies remain controversial. Most of the current literature is limited to small-scale studies. Systematic reviews have combined previously reported cases and discussed the features and prognostic factors for patients with MCT-SCC [7–9]. However, reporting bias is inherently inevitable since the inclusion of published data. Besides, the enrolled cases were diagnosed over the past 30 or 40 years, so many important factors have been changed substantially over such a long time. So the previous findings need to be investigated by a new study based on the latest population data.

The Surveillance Epidemiology and End Results (SEER) Program is a comprehensive and authoritative source of population-based information on cancer survival from registries that covers approximately 34.6% of the U.S. population [10]. The aim of this study was to understand the prognosis of ovarian SCC by using the most recent version of SEER database and analyzing the survivorship for patients with it or with SC, the most common histological type of epithelium ovarian cancer. Furthermore independent risk factors for poorer outcome for ovarian SCC were identified.

## Results

### The comparison of survival between patients with SC and SCC of ovary

**Fig. 1:**
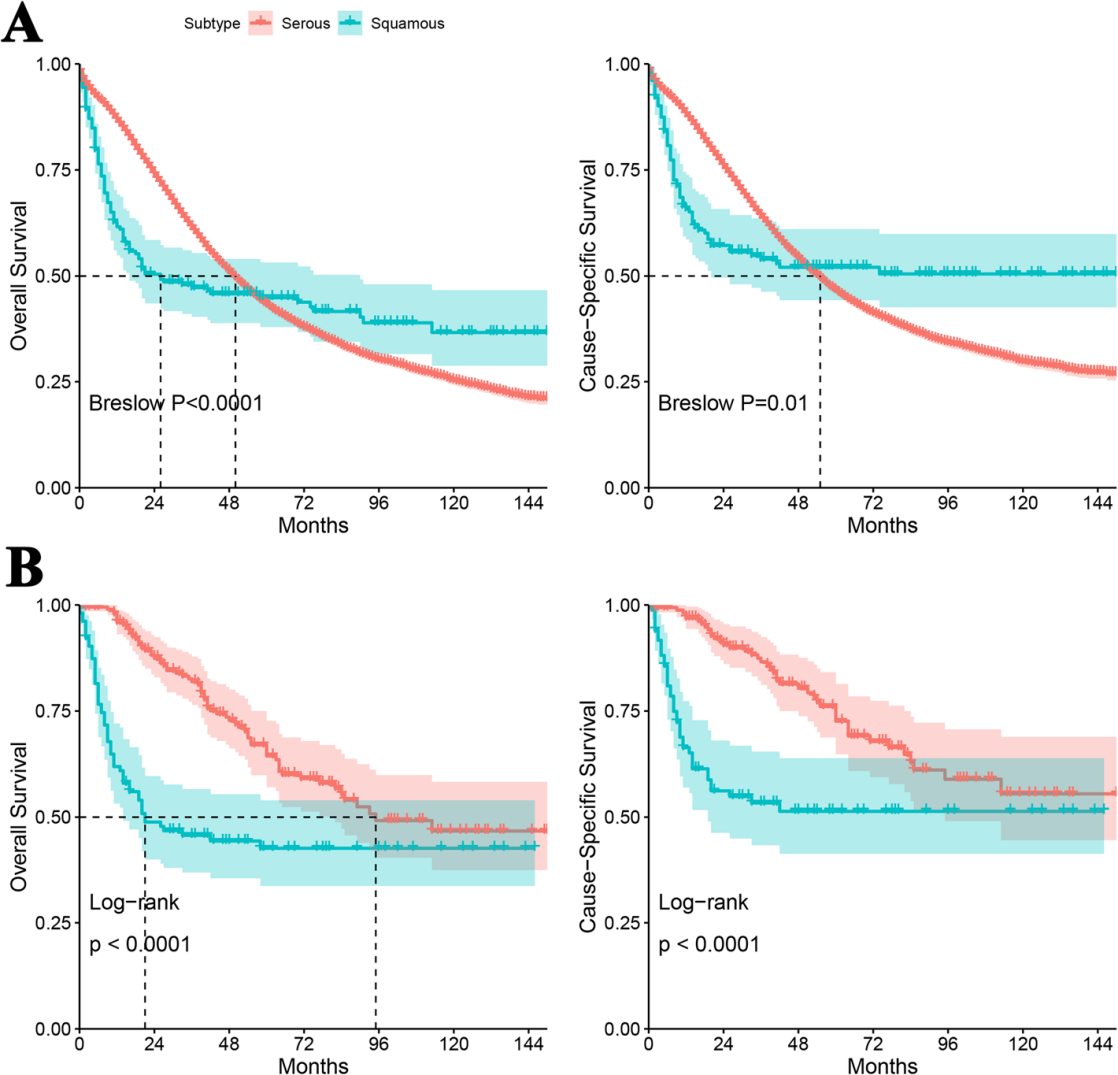
Survival plots compared the survival of patients with ovarian squamous cell carcinoma or with serous carcinoma. **a** The comparison before propensity score matching; **b** the comparison after propensity score matching

Regarding the patients with SC, the median OS and cause-specific survival time was 50.0 and 55.0 months, respectively (Fig. 1a). The 1-year, 2-year and 5-year OS rate was 86.8, 73.9 and 43.2%, respectively. The 1-year, 2-year and 5-year cause-specific survival rate was 88.1, 75.9 and 46.5%, respectively. Regarding the patients with SCC, the median OS time was 26.0 months (Fig. 1a). The median cause-specific survival time was not estimated since median survival was not reached. The 1-year, 2-year and 5-year OS rate was 61.6, 49.2 and 44.9%, respectively. The 1-year, 2-year and 5-year cause-specific survival rate was 65.8, 55.6 and 50.5%, respectively. Of note, the survival curves for SC and SCC crossed over, so breslow test was used (Fig. 1a, *P*_*breslow*_ < 0.05).

After 1:2 matching, the characteristics between the patients with SC and SCC were comparable (Table 1). Regarding the patients with SC, the median OS time was 95.0 months (Fig. 1b). The 1-year, 2-year and 5-year OS rate was 95.9, 87.6 and 64.4%, respectively. The 1-year, 2-year and 5-year cause-specific survival rate was 96.9, 90.7 and 72.6%, respectively. Regarding the patients with SCC, the median OS time was 21.0 months (Fig. 1b). The 1-year, 2-year and 5-year OS rate was 61.9, 47.8 and 42.6%, respectively. The 1-year, 2-year and 5-year cause-specific survival was 66.3, 54.8 and 51.4%, respectively. The survival plots showed the survival for patients with SCC was significantly worse than that for patients with SC (*P*_*log-rank*_ < 0.001).

**Table 1:** Baseline information for the patients with SCC and SC of ovary in the SEER database, N(%)

	Before matching			After matching		
	Serous (*n* = 15,106)	Squamous (*n* = 180)	*P*-value	Serous (*n* = 200)	Squamous (*n* = 104)	*P*-value
Age at diagnosis ^a^	62.0 (54.0–71.0)	55.5 (46.0–67.8)	< 0.001	58.5 (51.0–65.0)	57.0 (48.0–68.8)	0.745
Tumor size (mm) ^a^	79.0 (45.0–120.0)	90.0 (60.0–145.8)	0.003	95.5 (60.0–138.3)	90.0 (55.0–140.0)	0.727
Year of diagnosis						
≤2010	7094 (47.0)	107 (59.4)	0.001	108 (54.0)	57 (54.8)	0.893
> 2010	8012 (53.0)	73 (40.6)		92 (46.0)	47 (45.2)	
Race						
White	12,830 (84.9)	134 (74.4)	0.001	157 (78.5)	86 (82.7)	0.685
Black	1066 (7.1)	22 (12.2)		22 (11.0)	9 (8.7)	
Others	1169 (7.7)	23 (12.8)		21 (10.5)	9 (8.7)	
Unknown	41 (0.3)	1 (0.6)		–	–	
Laterality						
Unilateral	8609 (45.1)	172 (95.6)	< 0.001	194 (97.0)	101 (97.1)	0.955
Bilateral	8297 (54.9)	8 (4.4)		6 (3.0)	3 (2.9)	
FIGO stage						
I	1567 (10.4)	64 (35.6)	< 0.001	65 (32.5)	32 (30.8)	0.902
II	1348 (8.9)	39 (21.7)		46 (23.0)	30 (28.8)	
III	8092 (53.6)	44 (24.4)		60 (30.0)	26 (25.0)	
IV	4009 (27.1)	33 (18.3)		29 (14.5)	16 (15.4)	
Grade ^b^						
G1/G2	1690 (13.5)	60 (41.1)	< 0.001	46 (23.0)	32 (30.8)	0.141
G3/G4	10,837 (86.5)	86 (58.9)		154 (77.0)	72 (69.2)	
SEER stage						
Localized	833 (5.5)	47 (26.1)	< 0.001	32 (16.0)	22 (21.2)	0.448
Regional	2567 (17.0)	70 (38.9)		101 (50.5)	46 (44.2)	
Distant	11,706 (77.5)	63 (35.0)		67 (33.5)	36 (34.6)	

^a^ Data were described as median (P25 – P75). ^b^ The number of each stratum did not sum to the total number of cohort due to missing values

Abbreviations: *SCC* squamous cell carcinoma; *SC* serous carcinoma; *SEER* Surveillance Epidemiology and End Results Program; *FIGO* International Federation of Gynecology and Obstetrics

### The prognostic predictors for patients with SSC of ovary

To identify the risk factors for worse disease outcome, the cohort of 180 cases with SCC of ovary was further analyzed. The median time of follow-up was 20.0 months. One hundred and one (56.1%) and 70 patients (45.5%) were dead of any cause and specifically of SCC of ovary, respectively. As shown in Table 2, univariate Cox regression analyses showed that age, laterality, FIGO stage, SEER stage, lymphadenectomy and surgery influenced the OS and cause-specific survival (*P* < 0.05). Larger tumor size was only associated with worse OS (HR = 1.99, *P* = 0.034). Radiotherapy was only associated with worse cause-specific survival (HR = 1.75, *P* = 0.041).

**Table 2:** Univariate survival analyses for the patients with SSC of ovary in the SEER database (2004–2016, *N* = 180)

	Overall survival			Cause-specific survival		
Variables	HR	95%CI	*P*-value	HR	95%CI	*P*-value
Age						
≤67	1			1		
> 67	2.24	1.48–3.38	< 0.001	1.91	1.16–3.14	0.011
Race						
White	1			1		
Black	1.08	0.60–1.94	0.798	0.85	0.39–1.86	0.677
Others	0.86	0.48–1.62	0.638	0.96	0.47–1.94	0.901
Tumor size (mm)						
< 50	1			1		
50–150	1.99	1.05–3.75	0.034	2.23	0.99–5.07	0.054
> 150	1.75	0.82–3.74	0.150	2.12	0.84–5.32	0.110
Laterality						
Unilateral	1			1		
Bilateral	2.83	1.37–5.85	0.005	3.33	1.44–7.70	0.005
Serum CA125						
Negative	1			1		
Positive	1.42	0.78–2.57	0.248	1.82	0.86–3.83	0.116
FIGO stage						
I	1			1		
II	2.62	1.43–4.79	0.002	4.06	1.93–8.54	< 0.001
III	2.99	1.66–5.36	< 0.001	3.30	1.54–7.06	0.002
IV	9.56	5.27–17.36	< 0.001	16.52	7.86–34.72	< 0.001
Grade						
G1/G2	1					
G3/G4	1.23	0.78–1.92	0.371	1.18	0.70–2.02	0.533
SEER stage						
Localized	1			1		
Regional	2.62	1.37–5.03	0.004	4.27	1.77–10.33	0.001
Distant	6.40	3.38–12.1	< 0.001	9.70	4.06–23.15	< 0.001
Lymphadenectomy						
Yes	1			1		
No	1.67	1.11–2.50	0.014	1.70	1.04–2.78	0.034
Radiotherapy						
Yes	1			1		
No	0.76	0.47–1.22	0.254	0.57	0.33–0.98	0.041
Chemotherapy						
Yes	1			1		
No	1.01	0.68–1.49	0.964	1.21	0.75–1.94	0.437
Surgery						
Yes	1					
No	2.80	1.49–5.25	0.001	3.14	1.36–7.27	0.007

Abbreviations: *SCC* Squamous cell carcinoma; *SEER* Surveillance Epidemiology and End Results Program; *FIGO* International Federation of Gynecology and Obstetrics

**Fig. 2:**
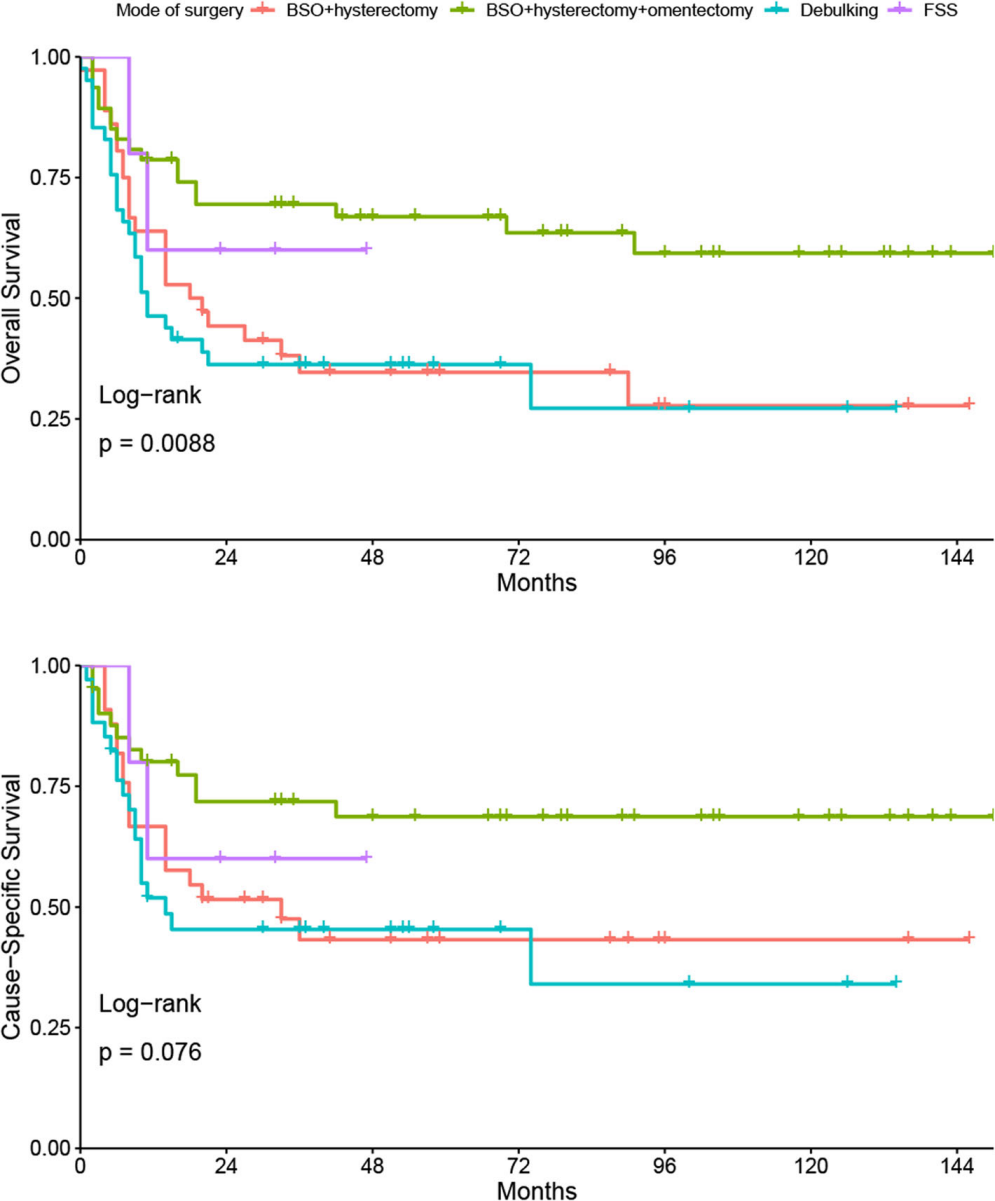
Survival plots compared the survival of patients with ovarian squamous cell carcinoma who underwent different modes of surgery

As far as the impact of surgery strategy was concerned, patients who underwent surgical treatment were analyzed. The survival plots showed that mode of surgery was associated with OS (*P*_*log-rank*_ = 0.0088, Fig. 2). Pairwise comparisons revealed significantly different OS in the comparison of debulking with bilateral salpingo-oophorectomy (BSO) + hysterectomy + omentectomy and the comparison of BSO + hysterectomy with BSO + hysterectomy + omentectomy. The bonferroni-corrected *p*-value was 0.042 and 0.012 for the two comparisons, respectively. The FDR-corrected p-value was 0.021 and 0.012, respectively. The OSs for FSS and radical surgery were comparable. Mode of surgery was not associated with cause-specific survival for patients who received surgery.

In the multivariate regression model, older age, larger tumor size, bilaterality (only for OS), FIGO stage IV were the independent risk factors for poor disease outcome. The hazard ratio increased with the tumor size category. For details, see Table 3.

**Table 3:** Multivariate survival analyses for the patients with SSC of ovary in the SEER database (2004–2016, *N* = 180)

	Overall survival			Cause-specific survival		
Variables	HR	95%CI	*P*-value	HR	95%CI	*P*-value
Age						
≤67	1			1		
> 67	6.77	2.55–18.01	< 0.001	6.39	1.84–22.25	0.004
Tumor size (mm)						
< 50	1			1		
50–150	5.17	1.14–23.49	0.033	7.07	0.81–61.35	0.076
> 150	7.98	1.47–43.42	0.016	12.00	1.21–119.19	0.034
Laterality						
Unilateral	1			1		
Bilateral	4.01	1.03–15.56	0.045	3.76	0.73–19.38	0.113
FIGO stage						
I	1			1		
II	1.15	0.25–5.25	0.855	0.82	0.14–4.79	0.823
III	2.86	0.82–9.92	0.099	1.83	0.43–7.91	0.416
IV	8.73	2.50–30.46	0.001	9.17	2.32–36.26	0.002

Variables in Cox regression models included: serum CA125, grade, surgery, lymphadenectomy, radiotherapy, and chemotherapy

Abbreviations: *SCC* Squamous cell carcinoma; *SEER* Surveillance Epidemiology and End Results Program; *FIGO* International Federation of Gynecology and Obstetrics

## Discussion

Ovarian SCC is a rare disease with reportedly poor prognosis. The 2-year and 5-year OS rate was 53.0 and 48.4% for MCT-SCC, respectively [7], however no direct comparison of survival between SCC and other ovarian cancers exist. Surprisingly, indirect comparison with the statistics provided by American Cancer Society (ACS) [1, 2] shows that the outcome of overall ovarian SCC is seemingly to be similar with the general or common subtypes of ovarian cancer. We directly compared the prognosis between ovarian SC and SCC by identifying cases who were diagnosed in the same period of time from the SEER database. The median survival time of SCC (26 months) was nearly halved than that of SC (50 months). The survival plot showed that the curve of SCC dropped rapidly during the first 2 years after diagnosis and became stable thereafter. The curve of SC fell smoothly. So the curves of SCC and SC split up initially and then crossed over. The 1-year and 2-year survival of SCC were substantially poorer, but the 5-year survival was slightly better, compared with SC. The 5-year cause-specific survival of SC was 46.5%, which was in line with the report from ACS (43%) [2].

Since the number of cases with SC dramatically overwhelmed and the features of SCC and SC were significantly different, we used 1:2 propensity score matching to identify cases with good comparability. After matching, the median survival time of SC (95 months) was about 4.5-fold longer than that of SCC (21 months). The 1-year, 2-year and 5-year survival of SCC were all worse than SC. To our best knowledge it is the first time to directly prove that ovarian SCC has a worse prognosis than other ovarian cancers. The survival curve also dropped fast at first for SCC, but it did not cross over the curve of SC eventually. Therefore we assumed that the intersection of the two curves before matching was partly due to the incomparable characteristics of the two groups. In addition, the intersected curve pattern might reflect the heterogeneity of disease origin. The pure primary ovarian SCC is extremely rare [11, 12], this disease is more likely to be metastases or arises from MCT, endometriosis, or Brenner tumors [6], some of which have particularly worse outcome. Metastatic ovarian SCC in cervical cancer may occur with advanced stage cervical carcinoma [13]. The 5-year survival for all-stage invasive cervical was about 65% [1, 14], and patients with stage III/IV had higher mortality (HR up to 7.4) [14]. The disease outcome of ovarian SCC originated from endometriosis was also extremely poor [15, 16]. Most of the cases were diagnosed with advanced stage and up to 80% of cases died within 6 months after diagnosis [16]. It is a limitation that we could not distinguish between ovarian SCCs arising from MCT, endometriosis, Brenner tumor and pure SCCs according to the SEER database, nor estimate the frequency of each subtypes based on current literature. Li et al. [9] enrolled 435 cases with MCT-SCC between 1977 and 2016. At the same time, Koufopoulos et al. [12] reviewed 36 cases with primary ovarian SCC before 2018. The SCCs arising from other lesions are sporadically reported. In this respect, we assume that the majority of ovarian SCC originates from malignant transformation of MCT.

The outcome of ovarian cancer is strongly determined by the stage at diagnosis. It is almost a consensus that advanced stage independently predicts worse outcome for patients with MCT-SCC [7–9]. In this study we also found that FIGO stage is an independent predictive factor for OS and cause-specific survival of ovarian SCC. In the multivariate model, the risks of overall death and cause-specific death for patients with FIGO stage IV increased by over 7–8 folds than patients with FIGO stage I. In addition, older age at diagnosis, larger tumor size and bilateral malignancy were independently associated with adverse outcome in this study, however different results have been also reported elsewhere. The review by Chen et al. [7] did not identify age, tumor size and laterality as independent factors by multivariate analysis. The review by Hackethal et al. [8] found tumor size was positively associated with age (*r* = 0.173), but the impact of them on survival was not investigated. The review by Li et al. [9] reported tumor size was not associated with disease outcome, and did not estimate the effect of laterality. So our findings should be treated with caution and confirmed by further studies.

According to the 2014 Gynecologic Cancer InterGroup (GCIG) consensus, there are very few information to provide widely-accepted guidelines for the management of this malignancy [6]. According to the evidences provided by systematic reviews, optimal debulking [7], hysterectomy [9] and omentectomy [9] have shown beneficial effect. In line with that, we found the disease outcome was generally better if surgical treatment was taken. Mode of surgery influenced the prognosis. It was shown that disease outcome was better if omentectomy had been performed. The 2014 GCIG consensus stated that surgical resection should be performed and complete cytoreduction should be the aim of surgery [6]. Total abdominal hysterectomy, BSO, and omentectomy are generally accepted [6]. The prognosis for patients who underwent FSS was comparable to patients who underwent radical surgery in this study. All cases received FSS were young (median age of 37) and were diagnosed with unilateral, FIGO stage I or II malignancy (data not shown). Likewise, Li et al. [9] demonstrated that there was no difference in outcome between FSS and radical surgery for patients younger than 45 years old with stage Ia or Ic. Thus FSS should be reserved to accurately selected young women with early-stage disease wishing to preserve fertility. For stage-specific groups, the results were more complex and inconsistent because disease stage and therapy strategy may interactively impact on the prognosis. Unfortunately there were insufficient data to further analyze the impact of different modes on groups with specific features in this study. It was suggested that MCT-SCC patients with tumor stage greater than FIGO Ia who had hysterectomy had longer survival time than those who did not receive this surgery [8]. In patients with stage II and above disease, omentectomy was not associated with improved survival whereas lymphadenectomy was [8].

The efficacy of radiotherapy and chemotherapy is still unclear. Although squamous cancers are generally sensitive to radiotherapy, no benefit was shown for SCC of ovary postoperatively [8, 9]. One study reported that surgery with adjuvant radiotherapy was superior to surgery alone for patients with stage III disease exclusively [7]. We found a marginally significant association (*P* = 0.041) of radiotherapy with worse survival for all-stage patients in univariate model, but this association turned insignificant in multivariate model. Regarding chemotherapy, it improved the outcome in patients with advanced stage disease [7, 9], and platinum-based regimen was related to better prognosis compared with other drugs [9]. Hackethal et al. [8] found that regimens with alkylating drugs were associated with increased survival in tumor stages greater than Ia. We failed to observe the beneficial effect of chemotherapy either in univariate or multivariate analysis. Moreover, we failed to analyze the efficacy of radiotherapy and chemotherapy for patients with specific stage due to the unavailability of information from the SEER database. The impact of cycle and regimen of chemotherapy was not taken in consideration either.

Although the patients with ovarian SCC may benefit from the current treatment, little improvement has been made in recent years, so it is vital to investigate novel agents and therapy. The sequencing experiment that covered 151 cancer-associated genes revealed that MCT-SCC had high mutation burden and shared similar mutation profile of lung SCC [17]. Therefore the author believed that patients with MCT-SCC could be included in trials of SCC-specific therapy and may benefit from immune checkpoint inhibition therapy like lung SCC [17]. Interestingly, one of the immune checkpoints, PD-L1, was found to be expressed on MCT-SCC tumor cells and was correlated with X-C motif chemokine ligand 1 (XCL1) expression and intratumor infiltration of CD8-positve T cells [18]. In this paper, it was hypothesized that the PD-L1/PD1 interaction mediated the dysfunction of cytotoxic T cell activated by XCL-1 ligation, so XCL1 might be new candidates for malignant transformation prediction and therapy response evaluation [18].

Considering the poor prognosis of this disease, early detection is of importance. In the present study, serum CA125 in prior to treatment was elevated in more than two thirds of the patients, which was in line with the previous reports [7–9, 19]. Since ovarian SCC is often accidentally found at surgery, pretreatment measure of serum tumor markers may be useful for the early detection of this malignancy. Apart from CA125, increased levels of SCC antigen [7–9, 19–22], macrophage colony-stimulating factor (M-CSF) [22], CA19–9 [7–9, 19] and carcinoembryonic antigen (CEA) [7–9, 19] were also suggested in literature. These tumor markers are promising for preoperative diagnosis of ovarian SCC. In addition to serological indicators, ultrasound imaging was also useful in differentiating malignant MCT-SCC from benign MCT [23]. Regarding prognosis prediction, elevated CA125 was not a risk factor for worse outcome in the present study. Interestingly, the two large-scale reviews [7, 8] demonstrated CA125 as a prognostic factor for MCT-SCC in univariate model, but did not examine its efficacy in Cox regression model. For other ovarian cancers, it has been indicated that CA125 was a valuable predictor in univariate model but lost its significance when FIGO stage was considered in multivariate model [24, 25]. So it is still controversial whether preoperative serum CA125 is an independent predictor for ovarian SCC outcome.

The data is limited to America, information from the rest of the world are needed. For example, there is no cohort for this specific rarity in China mainland, and such cases are sporadically documented in case report or case series [9, 16, 26–28]. Due to the low incidence of the disease, widespread collaboration across multiple centers may establish a cohort study with a relatively larger sample size. For a single center, it usually takes 6–8 years to identify 1 case [29, 30]. For multiple centers, The Taiwanese Gynecologic Oncology Group (TGOG) study retrospectively reviewed 24,040 patients with MCT and 16,001 patients with primary ovarian cancer from 10 medical centers over 21 years and finally identified 52 patients with MCT-SCC [31]. So there is still a long way to go before a national cohort of ovarian SCC is established. We hope this study could contribute to our current knowledge about this rarity by using the latest population database.

## Conclusion

The prognosis of ovarian SCC is worse than ovarian SC. Older age at diagnosis, advanced disease stage, larger tumor size and bilateral tumor are the independent risk factors for poor survival of ovarian SCC. There are few information to provide guidance on the optimal therapy strategy.

## Methods

### Data source and case selection

Patients with SCC and SC were identified using the recent SEER database released in Nov 2018 [32]. The dataset was accessed using SEER*Stat software 8.3.6 (National Cancer Institute, USA) with the permission from the SEER office. The site specific International Classification of Diseases for Oncology-3 (ICD-O-3) code of C56.9 was used to identify ovarian cancer. The behavior ICD-O-3 code was 3 (malignant). The histologic type ICD-O-3 code of 8070 was used to identify CSS and the codes of 8441, 8461 and 9014 were used the identify SC. Cases who were diagnosed from 2004 to 2016 were included. No other restrictions were imposed.

The following variables were obtained from the database: patient ID, race, age at diagnosis, vital status, cause-specific death, survival months, laterality, grade, TNM stage (based on 7th edition), SEER stage, surgical treatment, radiotherapy, chemotherapy, lymphadenectomy, tumor size and serum carbohydrate antigen 125 (CA125) prior to treatment. FIGO stage was assessed based on TNM stage. Serum CA125 was recorded as elevated (positive), within normal limits (negative) prior to treatment or test undone (blank value). The mode of surgery was re-coded as no surgery, BSO with hysterectomy, BSO with hysterectomy and omentectomy, fertility-sparing surgery (FSS) or debulking.

### The characteristics of participants

**Fig. 3:**
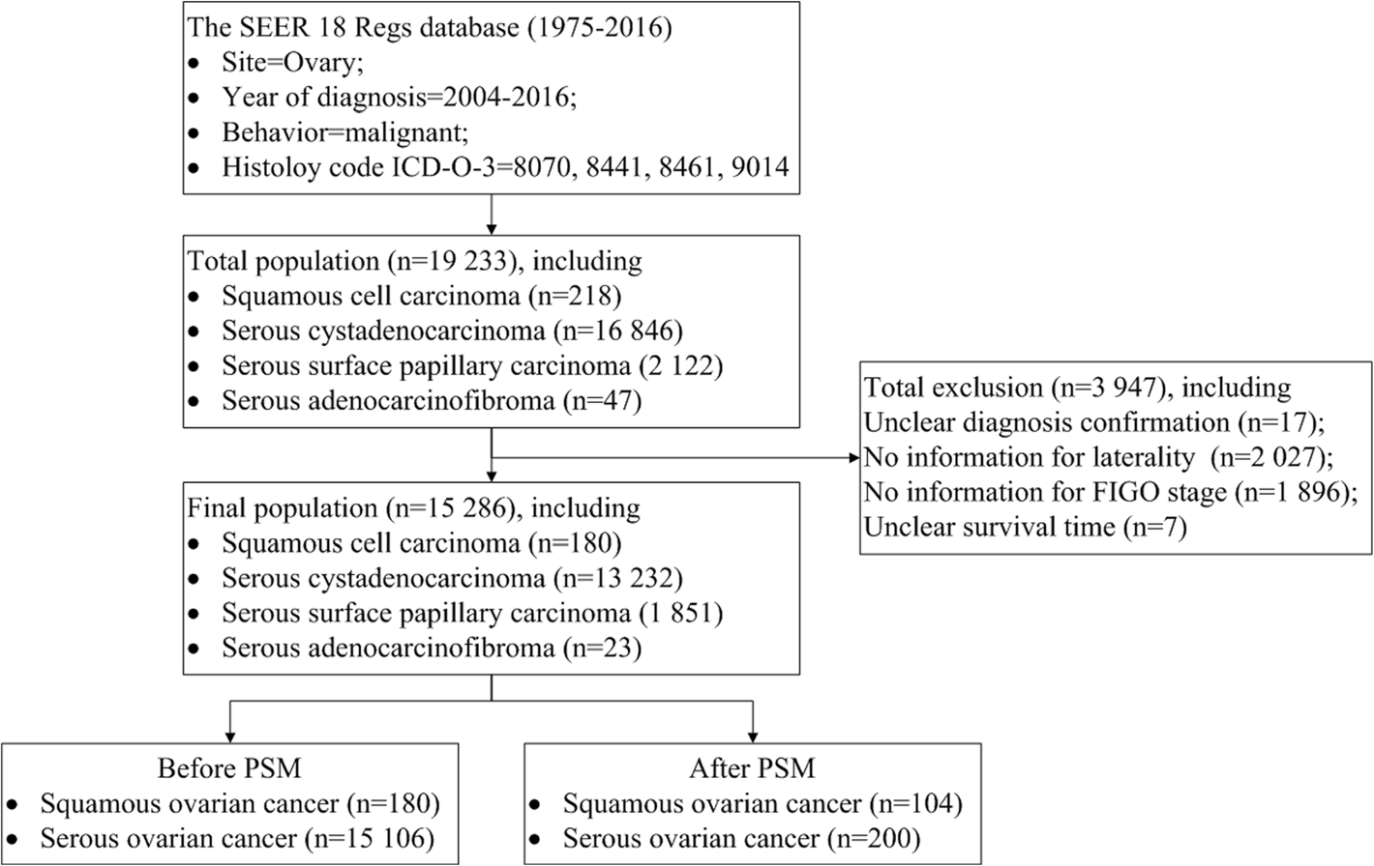
Flow diagram of case selection

A total of 19,233 cases were selected. After excluding cases with no information for critical variables (*N* = 3947), 15,286 patients were finally included in the analysis, among whom 15,106 patients were diagnosed with SC and 180 patients were diagnosed with SCC. The flow diagram was shown in Fig. 3. The detailed baseline information was shown in Table 3. Briefly for demographic features, more patients with SCC were not white, diagnosed at younger age and before 2010 than patients with SC were. For clinical characteristics, patients with SCC were more likely to be diagnosed with unilateral, lower FIGO stage, well-differentiated and localized malignancies than patients with SC were.

### Statistical analyses

Demographic and clinicalpathological characteristics were compared between cases with SCC and SC. The optimum cut-off value for transformation of continuous variable into categorical variable was determined using X-tile software version 3.6.1 [33]. Univariate associations were analyzed using chi-square test for categorical variables or using Kruskal-Wallis test for ordinal variables. The Mann-Whitney U test was performed to identify significant difference in quantitative variable. Univariate and multivariate Cox regression analyses were used to evaluate the impact of variables on the OS and cause-specific survival of patients. OS or cause-specific survival were defined as the time (in months) from diagnosis to death of any cause or specifically of ovarian SCC, respectively. The survival plot was made using Kaplan-Meier method and compared using breslow or log-rank test. The *p*-values of pairwise log-rank tests were corrected by “bonferroni” and “FDR” methods using “p.adjust” syntax of R program [34]. All statistical analyses were performed with Rstudio version 1.1456. A p-value less than 0.05 was considered as statistically significant.

Since the data was from national cancer registry project, no control over serous and SCC patients was assigned. Therefore, large differences on observed covariates in the two groups might exist. Besides, since ovarian SCC is rare, there were a limited number of such patients and a much larger number of serous patients. Both of the two issues could lead to biased estimates. Matching is a common technique used to select control subjects and balance the covariates in the two groups. In the present study, the propensity score for an individual, which considered many background covariates, was used for matching as a single scalar variable. Here this score was defined as the likelihood of being ovarian SC predicted by a logistic regression model. The background covariates it considered were as follows: race, year of diagnosis, age at diagnosis, tumor size, laterality, FIGO stage, SEER stage and histological grade. Since the number of controls (SC patients) was huge, individuals were matched 1:2 into SCC and SC groups, thus obtaining a larger sample size and a relatively more robust and powerful result. The method was the nearest neighbor matching with a caliper of 0.02 using “MatchIt” package of R program [35]. No replacement was allowed, and patients were matched only once.

## Data Availability

The datasets analyzed during the current study are available in the SEER database.
